# Comprehensive Evaluation and DNA Fingerprints of *Liriodendron* Germplasm Accessions Based on Phenotypic Traits and SNP Markers

**DOI:** 10.3390/plants14172626

**Published:** 2025-08-23

**Authors:** Heyang Yuan, Tangrui Zhao, Xiao Liu, Yanli Cheng, Fengchao Zhang, Xi Chen, Huogen Li

**Affiliations:** 1 State Key Laboratory of Tree Genetics and Breeding, Co-Innovation Center for Sustainable Forestry in Southern China, Nanjing Forestry University, Nanjing 210037, China; hyyuan@njfu.edu.cn (H.Y.); terryzhao@njfu.edu.cn (T.Z.);; 2 College of Architecture, Anhui Science and Technology University, Bengbu 233100, China

**Keywords:** *Liriodendron*, germplasm evaluation, genetic diversity, SNP, DNA fingerprinting

## Abstract

Germplasm resources embody the genetic diversity of plants and form the foundation for breeding and the ongoing improvement of elite cultivars. The establishment of germplasm banks, along with their systematic evaluation, constitutes a critical step toward the conservation, sustainable use, and innovative utilization of these resources. *Liriodendron*, a rare and endangered tree genus with species distributed in both East Asia and North America, holds considerable ecological, ornamental, and economic significance. However, a standardized evaluation system for *Liriodendron* germplasm remains unavailable. In this study, 297 *Liriodendron* germplasm accessions were comprehensively evaluated using 34 phenotypic traits and whole-genome resequencing data. Substantial variation was observed in most phenotypic traits, with significant correlations identified among several characteristics. Cluster analysis based on phenotypic data grouped the accessions into three distinct clusters, each exhibiting unique distribution patterns. This classification was further supported by principal component analysis (PCA), which effectively captured the underlying variation among accessions. These phenotypic groupings demonstrated high consistency with subsequent population structure analysis based on SNP markers (K = 3). Notably, several key traits exhibited significant divergence (*p* < 0.05) among distinct genetic clusters, thereby validating the coordinated association between phenotypic variation and molecular markers. Genetic diversity and population structure were assessed using 4204 high-quality single-nucleotide polymorphism (SNP) markers obtained through stringent filtering. The results indicated that the *Liriodendron sino-americanum* displayed the highest genetic diversity, with an expected heterozygosity (He) of 0.18 and a polymorphic information content (PIC) of 0.14. In addition, both hierarchical clustering and PCA revealed clear population differentiation among the accessions. Association analysis between three phenotypic traits (DBH, annual height increment, and branch number) and SNPs identified 25 highly significant SNP loci (*p* < 0.01). Of particular interest, the branch number-associated locus SNP_17_69375264 (*p* = 1.03 × 10^−5^) demonstrated the strongest association, highlighting distinct genetic regulation patterns among different growth traits. A minimal set of 13 core SNP markers was subsequently used to construct unique DNA fingerprints for all 297 accessions. In conclusion, this study systematically characterized phenotypic traits in *Liriodendron*, identified high-quality and core SNPs, and established correlations between key phenotypic and molecular markers. These achievements enabled differential analysis and genetic diversity assessment of *Liriodendron* germplasm, along with the construction of DNA fingerprint profiles. The results provide crucial theoretical basis and technical support for germplasm conservation, accurate identification, and utilization of *Liriodendron* resources, while offering significant practical value for variety selection, reproduction and commercial applications of this species.

## 1. Introduction

The genus *Liriodendron*, belonging to the family Magnoliaceae, comprises two extant species: *Liriodendron chinense*, native to East Asia, and *Liriodendron tulipifera*, found in eastern North America. *Liriodendron* species are large deciduous trees that can grow up to 40 m tall and are characterized by their distinctive leaf shapes, showy flowers, and straight, upright trunks—traits that contribute to their ecological, ornamental, and timber value. The wood is lightweight, fine-textured, and naturally resistant to pests, while the trees themselves demonstrate strong environmental adaptability, including tolerance to air pollution [1]. Owing to their ecological resilience and economic utility, *Liriodendron* species hold significant potential for research on phylogeny, genetic diversity, and species conservation, as well as for breeding and practical applications in forestry and landscaping [2].

The comprehensive collection, preservation, and assessment of *Liriodendron* germplasm resources serve dual critical purposes of safeguarding genetic diversity and ecological functionality while establishing the essential foundation for breeding superior cultivars through contemporary forest tree improvement programs. Phenotypic characterization represents the most immediate and reliable methodology for germplasm evaluation, enabling thorough documentation of accession performance and revealing underlying genetic diversity and adaptive potential [3]. Recent investigations have yielded systematic advances in understanding key phenotypic attributes of *L. tulipifera*, *L. chinense*, and their interspecific hybrids. Notably, Zong et al. identified three AP2/ERF transcription factors exhibiting shoot apical meristem-specific expression patterns in *Liriodendron* through genome-wide analysis, potentially governing early leaf morphogenesis [4]. Significant progress has also been made in flowering trait research, with Sheng et al. elucidating floral transition regulatory mechanisms via comparative transcriptomic profiling [5]. Furthermore, Liu et al. characterized spatiotemporal expression dynamics among MADS-box transcription factors during floral development, providing mechanistic insights into floral architecture variation within the genus [6]. These meticulous phenotypic analyses have significantly advanced our comprehension of phenotypic plasticity and adaptive evolutionary processes in *Liriodendron*, while simultaneously informing practical applications in germplasm classification, conservation management, and genetic enhancement initiatives. Although many researchers have conducted extensive studies on phenotypic traits and genetic mechanisms in *Liriodendron* [7,8,9], the integrated evaluation systems combining phenotypic traits with SNP markers remains underdeveloped.

Evaluating and characterizing the genetic diversity of germplasm resources is essential for constructing core germplasm collections. To date, a variety of molecular markers have been utilized in plant genetic research, including conventional markers such as restriction fragment length polymorphism (RFLP), random amplified polymorphic DNA (RAPD), amplified fragment length polymorphism (AFLP), inter-simple sequence repeat (ISSR), and simple sequence repeat (SSR), as well as sequencing-based markers such as single nucleotide polymorphisms (SNPs) and insertion/deletion polymorphisms (InDels) [10]. Recent advances in high-throughput sequencing technologies have significantly enhanced association analyses between molecular markers and plant phenotypic traits, establishing this approach as a powerful methodology for elucidating genetic diversity and developing comprehensive germplasm resource maps. Representative studies demonstrate the effectiveness of this strategy. Wang et al. successfully identified multiple SCoT marker loci significantly associated with 12 ornamental traits through marker-trait association analysis of 65 chrysanthemum germplasm accessions [11]. In parallel research, Donkpegan et al. performed genome-wide association analysis on 23 fruit quality traits across 116 sweet cherry germplasm resources, pinpointing SNP markers strongly correlated with critical agronomic characteristics including fruit size and firmness [12]. These investigations collectively provide molecular-level insights into phenotypic expression patterns. In phenotypic–molecular association studies, SNPs have become the predominant marker class owing to their abundance, genome-wide coverage, and high information density [13]. Their effectiveness has been demonstrated across diverse taxa, including *Acorus tatarinowii* [14], *Zea mays* [15], and *Dioscorea rotundata* [16], in which these markers have proved useful for assessing genetic diversity and constructing DNA fingerprints.

With the advancement of DNA molecular marker technology, the evaluation of germplasm resources in an increasing number of species has shifted from phenotypic characterization to high-resolution genotyping, providing a foundation for the development of standardized DNA fingerprinting systems. In recent years, SNP-based and SSR-based fingerprinting platforms have been established in most tree species [17]. For example, Yan et al. [18] analyzed the genetic diversity and genetic structure of 161 clonal lines of *Pinus koraiensis* using SSR markers and successfully constructed a robust DNA fingerprinting system. Similar studies have also been conducted in *Ailanthus altissima* [19] and *Camellia sinensis* [20]. This technique has been widely applied across various taxa, including vegetables (e.g., *Raphanus sativus* [21], *Brassica oleracea* var. *botrytis* [22], *Ipomoea batatas* [23]) and fruits (e.g., *Vaccinium corymbosum* [24], *Morus alba* [25], *Prunus avium* [26]). However, to date, there is no efficient and high-resolution molecular identification system available for *Liriodendron* germplasm resources, which presents challenges for germplasm management, breeding, and intellectual property protection in this genus [27]. Therefore, establishing a comprehensive DNA fingerprinting platform for *Liriodendron* using high-throughput molecular markers has become a pressing research priority. As a fundamental tool for germplasm characterization, DNA fingerprinting enables the generation of unique molecular identifiers by detecting genomic variations with high specificity [19]. Compared with traditional phenotypic assessments, this approach offers significant advantages in accuracy, reproducibility, and scalability. The development of SNP-based DNA fingerprints for *Liriodendron* is thus of great significance for both theoretical research and practical applications in germplasm conservation and breeding programs.

This study utilized 297 *Liriodendron* germplasm accessions as experimental materials and performed systematic assessments of 34 phenotypic traits. Comprehensive analysis demonstrated considerable phenotypic variation in most evaluated traits, along with statistically significant inter-trait correlations. Based on high-throughput sequencing of 197 representative samples, a set of high-quality single-nucleotide polymorphism (SNP) markers was identified, from which core SNP markers were selected for downstream analysis. The resulting genome-wide SNP marker system enabled precise assessments of genetic diversity and genetic structure within the *Liriodendron* germplasm collection. Furthermore, a robust DNA fingerprinting platform with high discriminatory power was developed to support accurate resource authentication, digital archiving, and traceability. These methodological advances substantially improve the precision, efficiency, and reliability of *Liriodendron* germplasm conservation and utilization.

## 2. Results

### 2.1. Evaluation of Phenotypic Traits

#### 2.1.1. Analysis of Phenotypic Diversity in Populations

Analysis of 34 phenotypic traits across 297 *Liriodendron* accessions (Table 1) revealed substantial patterns of morphological diversity. Growth-related traits exhibited particularly high variation, with annual DBH increment showing the greatest diversity (Shannon index H′ = 5.23; CV = 26.57%), followed by height growth (H′ = 4.71; CV = 24.25%), indicating pronounced genetic variation in growth performance. Architectural traits demonstrated moderate to high variability, including crown width (H′ = 0.89; CV = 38.46%) as well as branch characteristics—branch number (H′ = 3.33; CV = 37.68%) and branch density (H′ = 1.07; CV = 40.85%). Interestingly, the branching pattern, despite being a qualitative trait, exhibited unexpectedly high diversity (H′ = 0.96; CV = 40.85%). Phenological traits showed consistent but comparatively lower levels of variation, such as leaf budburst (H′ = 1.05; CV = 33.88%) and flowering time (H′ = 0.91; CV = 30.68%). Foliar color traits displayed intermediate levels of diversity, comparable to those of structural characteristics.

#### 2.1.2. Phenotypic Trait Clustering and Correlation Analysis

**Figure 1:**
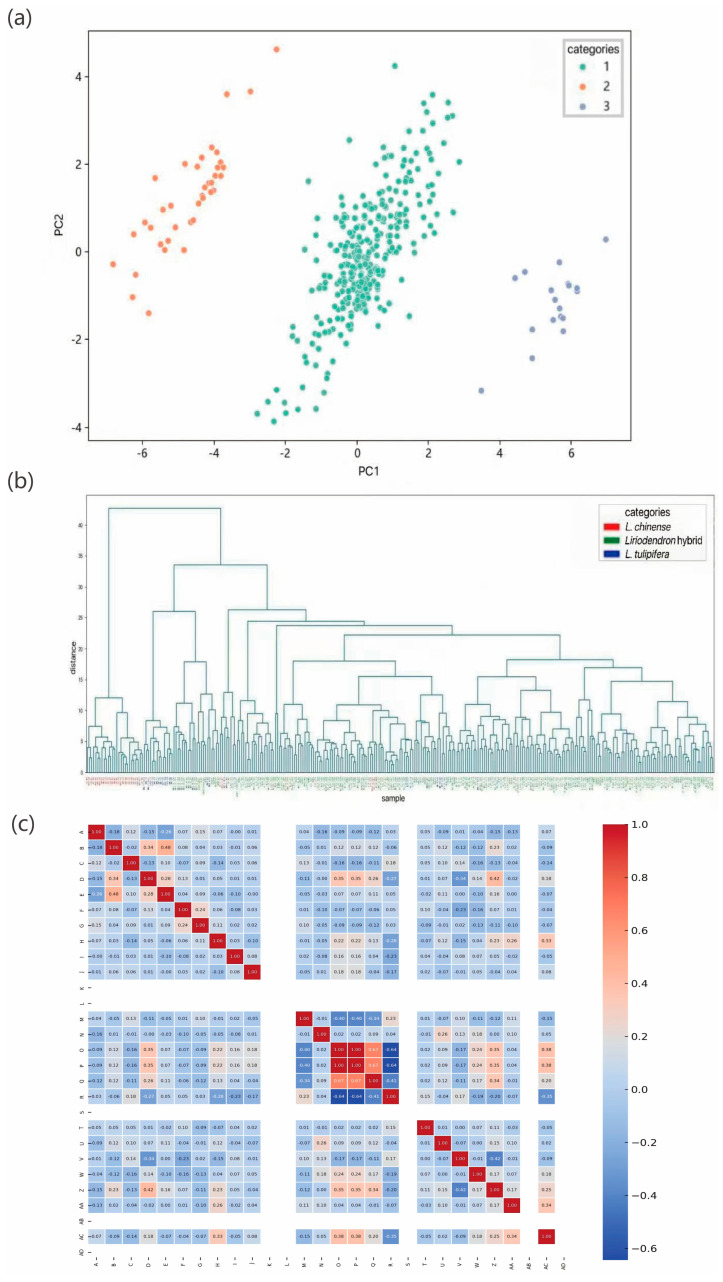
Multivariate phenotypic analysis of *Liriodendron* germplasm: (**a**) Principal Component Analysis (PCA) showing green, orange, and purple data points corresponding to *Liriodendron sino-americanum*, *L. tulipifera and L. chinense*; (**b**) Hierarchical clustering with blue, green and orange clusters representing *L. chinense, Liriodendron sino-americanum* and *L. tulipifera*; (**c**) Trait correlation matrix (Pearson’s r) with color gradient indicating correlation strength.

Principal component analysis (PCA) was conducted to reduce trait dimensionality and explore sample distribution patterns. The first two principal components (PC1 and PC2) accounted for 74.06% of the total phenotypic variance (Figure 1a), with samples clearly segregating into three distinct clusters. Hierarchical clustering (HC) analysis (Figure 1b) further supported this classification. *Liriodendron sino-americanum* individuals formed multiple branches that were partially intermixed with either *L. chinense* or *L. tulipifera* samples. *L. tulipifera* accessions clustered tightly within a separate branch, indicating strong genetic homogeneity. Most *L. chinense* samples were grouped within a single major cluster, reflecting a relatively conserved germplasm background with limited intra-population divergence. The sample groupings identified by HC were consistent with those from PCA, validating the robustness of the observed clustering pattern. These intergroup differences likely reflect underlying covariation among traits. To further investigate trait associations, Pearson correlation coefficients were calculated and visualized in a heatmap (Figure 1c).

The correlation analysis revealed three distinct patterns of phenotypic associations among the measured traits: (1) strong positive correlations were observed between specific trait combinations, including crown width and branch thickness, tree height and subbranch height, as well as branch density and crown width etc.; (2) significant negative correlations were identified between other trait pairs, such as crown shape and branch density, bark fissures and leaf bud break timing, and leaf abscission and autumn leaf coloration etc.; (3) weak or negligible correlations were observed between certain traits, such as crown width and bark coloration, as well as branch thickness and inner bark pigmentation. These findings collectively elucidate the comprehensive structure of phenotypic trait relationships.

### 2.2. SNP Marker Screening and Sanger Sequencing Validation

#### 2.2.1. Selection of SNP Markers

**Figure 2:**
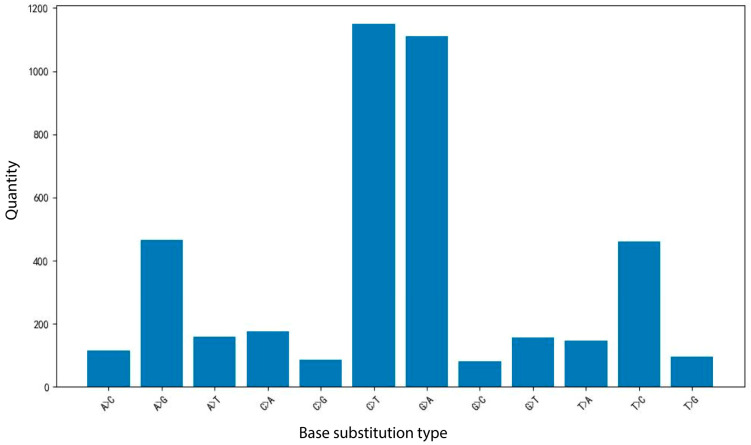
Distribution map of SNP base substitution variant types. As shown in the figure.

A total of 2165146 SNPs were identified using 197 whole-genome resequencing datasets. According to the filtering criteria, 4204 high-quality SNPs were ultimately obtained. In the statistics of SNP single-base substitution types, 12 types of variation were detected, indicating the presence of abundant variation types in the *Liriodendron* genome. Among them, 3189 transitions and 1015 transversions were detected, with a Ts/Tv ratio of 3.1419, indicating that the research data are highly reliable and consistent with the general characteristics of high-quality SNPs (Figure 2). Based on the screened high-quality SNPs, core SNPs were selected, resulting in 13 core SNP markers (Table 2) distributed across 8 chromosomes, ensuring broad genome coverage.

#### 2.2.2. Sanger Sequencing Validation

**Figure 3:**
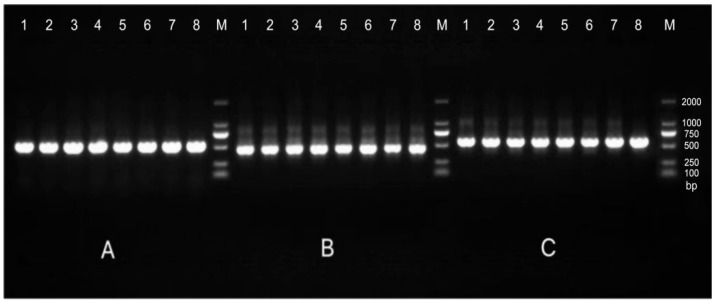
Representative agarose gel electrophoresis image validating 13 core SNP markers across eight *Liriodendron* accessions as shown in the figure. Lanes 1–8 represent eight independent samples, with DNA bands of the expected size. M represents the DNA marker (indicate size range, e.g., 100–2000 bp); (**A**–**C**) correspond to the first three primer pairs, with eight samples tested for each primer pair.

The accuracy and genotyping reliability of the selected core SNP markers were validated experimentally. Specific primers were designed for each of the 13 core SNP loci. Eight representative samples were selected for PCR amplification, followed by agarose gel electrophoresis to assess amplification efficiency. In addition, three samples representing distinct genotypes were chosen for Sanger sequencing at the Chr7:54379502 locus as an example of validation. The results (Figure 3) demonstrated that the designed primers exhibited high specificity and stability across all tested samples. The sequencing chromatograms (Table 3) showed clear and symmetrical peak patterns for all three genotypes, which were fully consistent with the genotyping results obtained from high-throughput sequencing. These results confirm the high accuracy and reliability of SNP genotyping.

### 2.3. Genetic Diversity Analysis

Genetic diversity indices were systematically compared among three groups: *Liriodendron sino-americanum*, *L. tulipifera*, and *L. chinense*. Significant differences were observed across multiple parameters (Table 4). The *Liriodendron sino-americanum* group exhibited the highest levels of genetic diversity, including the number of alleles (Na = 2.00), observed heterozygosity (Ho = 0.20), expected heterozygosity (He = 0.18), polymorphism information content (PIC = 0.14), Shannon’s diversity index (H′ = 0.46), and Nei’s genetic diversity index (Nei = 0.18), indicating a rich allelic composition and substantial genetic variation. *L. tulipifera* showed intermediate diversity levels, with relatively balanced values across all indices, suggesting a genetically stable background while retaining a moderate degree of variation. In contrast, *L. chinense* consistently exhibited the lowest diversity values (e.g., Na = 1.36; He = 0.13), reflecting a more conserved genetic structure. Notably, observed heterozygosity (Ho) was lower than expected heterozygosity (He) across all three groups, implying potential inbreeding or the influence of selection pressures. The elevated diversity in the hybrid population is likely due to parental gene admixture, whereas the reduced diversity in *L. chinense* may be attributed to its limited geographic distribution, small natural population size, long-term isolation, anthropogenic disturbances, and historical bottleneck events.

### 2.4. Genetic Differentiation: Clustering and Population Structure Analysis

**Figure 4:**
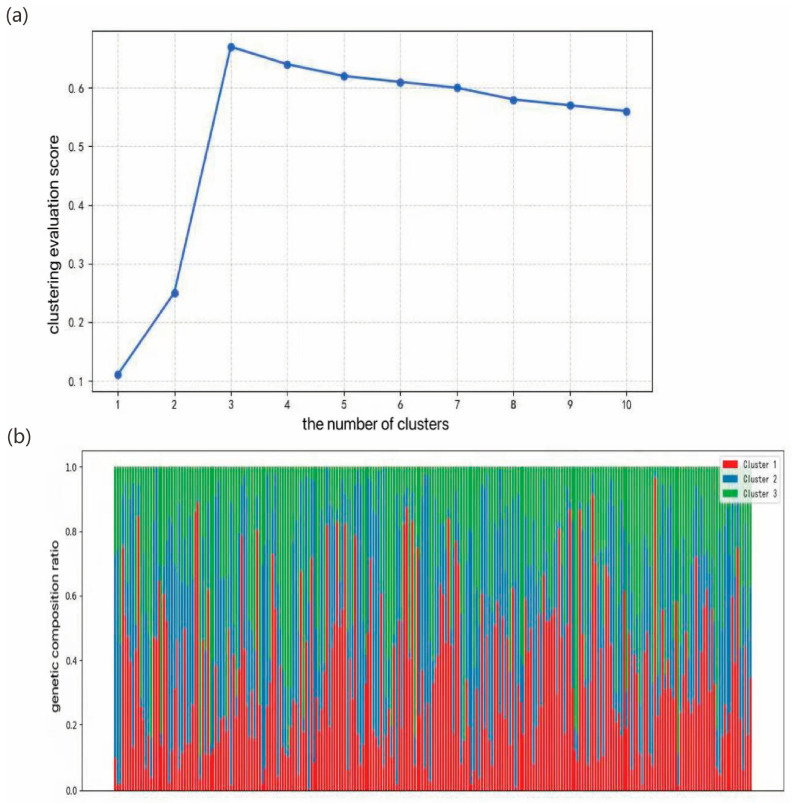
(**a**) Population structure clustering effect (Bayesian clustering model) score plot of 197 materials under different K values; (**b**) Genetic admixture proportions among 197 accessions at optimal K = 3. Color-coded clusters: Cluster 1 (red) = *L. tulipifera*, Cluster 2 (blue) = *L. chinense*, Cluster 3 (green) = *Liriodendron sino-americanum*.

The population structure of *Liriodendron* was analyzed using a Bayesian clustering model. Following the method of Evanno et al. [28], the optimal number of genetic clusters (K) was determined based on cross-validation scores from 197 samples (Figure 4a). When K = 3, the cross-validation error reached its minimum and ΔK achieved its maximum, indicating that the 197 accessions could be reliably partitioned into three genetic subpopulations. The population structure bar plot for K = 3 (Figure 4b) further illustrates the proportional genetic composition of each sample within the three inferred clusters. Several accessions displayed clear assignments to a single cluster, indicating strong genetic homogeneity, whereas most individuals exhibited admixture from two or all three clusters, reflecting their complex genetic backgrounds.

**Figure 5:**
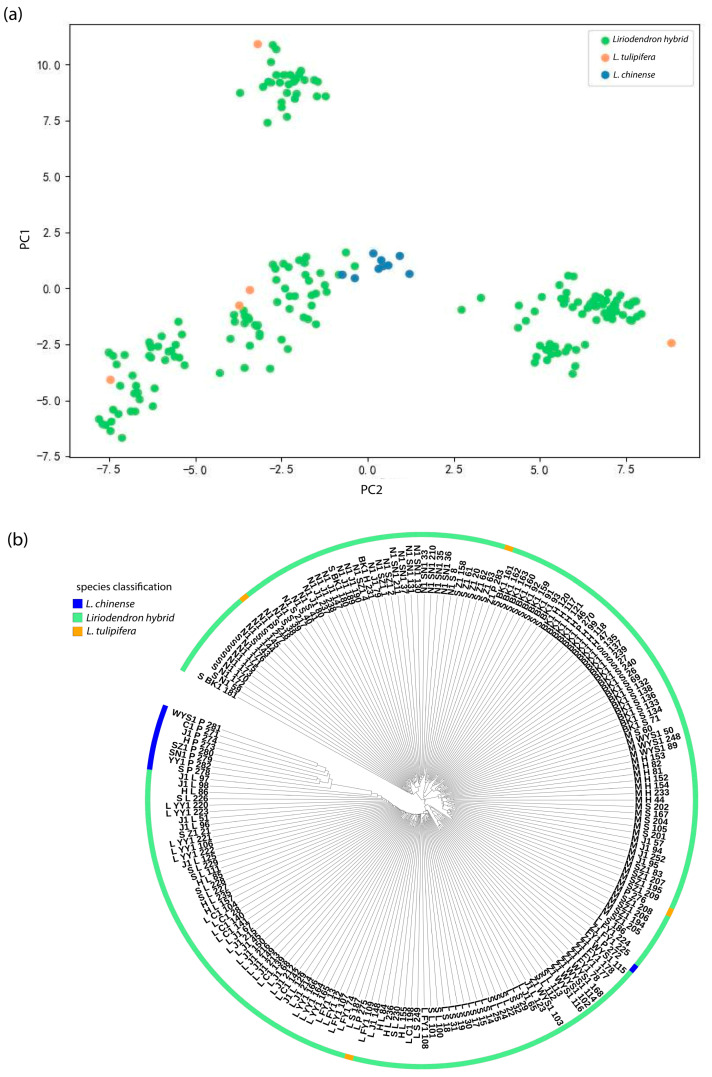
(**a**) Principal Component Analysis (PCA) based on SNP markers; (**b**) UPGMA clustering based on SNP markers. Color-coded representation of different *Liriodendron* groups (as shown in the figure).

Principal component analysis (PCA) of the genotype data was conducted to characterize the population genetic structure. The first two principal components explained 5.39% (PC1) and 3.57% (PC2) of the total genetic variation, yielding a cumulative explanatory power of 8.96%. This modest variance capture by the leading components principally stems from two biological factors: (i) pervasive gene flow maintaining genetic connectivity across the population, resulting in a continuous allele frequency distribution rather than discrete clusters, and (ii) the genome-wide random distribution of variation from the 4204 predominantly neutral SNPs employed in our analysis. Notably, despite the limited proportion of total variance explained, the two-dimensional PCA projection effectively resolved major axes of genetic differentiation among samples. The analysis divided the 197 accessions into three genetic groups. In the two-dimensional PCA plot (Figure 5a), *L. tulipifera* and *L. chinense* formed distinct clusters at opposite ends of the plot, confirming strong genetic differentiation between the two species. *Liriodendron sino-americanum* (*L. tulipifera × L. chinense*) individuals were positioned intermediately, with several samples overlapping those of *L. chinense*, indicating the primary direction of genetic divergence. Hierarchical clustering based on Euclidean genetic distances using the UPGMA algorithm produced results consistent with both the PCA and STRUCTURE analyses. The resulting dendrogram (Figure 5b) showed three well-defined genetic groups, with the majority of samples clustering within a major branch dominated by *Liriodendron sino-americanum*, highlighting their prevalence in the current germplasm collection. The molecular classification of these *Liriodendron* germplasm accessions showed high concordance with phenotypic groupings derived from principal component and cluster analyses, demonstrating significant associations between SNP markers and phenotypic traits.

### 2.5. SNP-Based Association Analysis of Phenotypic Traits

**Figure 6:**
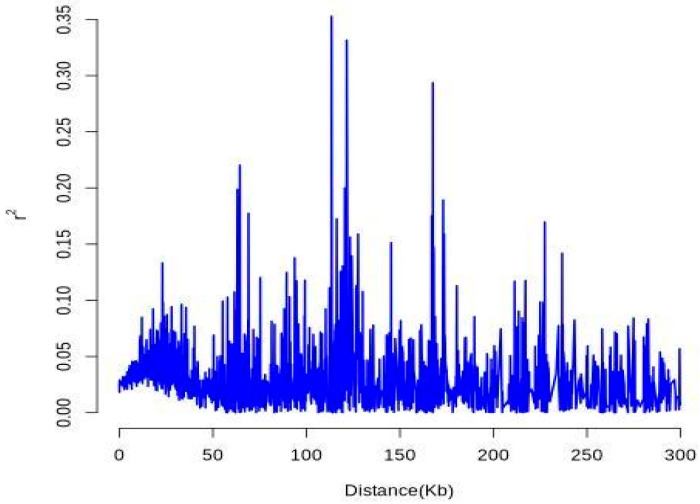
Linkage Disequilibrium (LD) decay plot.

SNP-based linkage disequilibrium (LD) analysis revealed relatively high *r*^2^ values at short physical distances (0–20 kb), followed by a rapid decay as distance increased, indicating fast LD decay within the population (Figure 6). This pattern suggests high recombination frequency and considerable genetic diversity. Multiple localized *r*^2^ peaks were detected across the genome, potentially corresponding to structural variants, loci under selection, or repetitive genomic regions.

**Figure 7:**
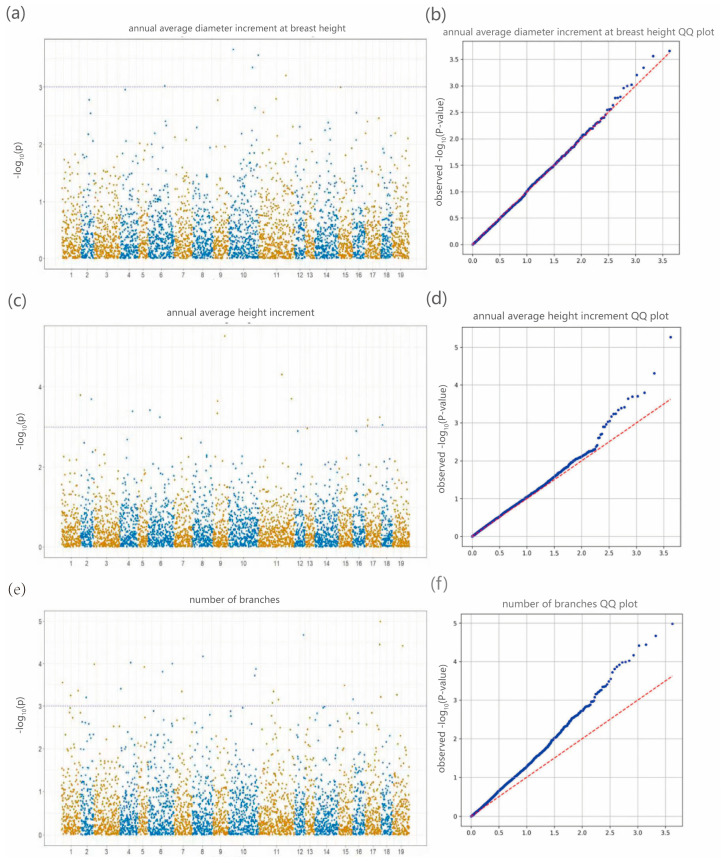
Single-Trait GWAS of Growth Traits in 197 *Liriodendron* Samples (**a**,**b**) Manhattan plot and QQ plot for the annual average diameter at breast height increment The black dashed line indicates the significance threshold at *p* = 1 × 10^−3^. In QQ plot, the red and blue dashed lines represent the expected distribution and the observed distribution, respectively. (**c**,**d**) Manhattan plot and QQ plot for the annual average height increment (**e**,**f**) Manhattan plot and QQ plot for the number of branches.

The GLM-based genome-wide association study (GWAS) of annual average diameter at breast height increment, annual height growth, and branch number revealed distinct patterns of SNP–trait associations. For branch number, multiple significant SNPs exceeded the significance threshold, with noticeable deviations from the expected distribution in the QQ plot. In contrast, the Manhattan plots for DBH and height growth displayed fewer pronounced peaks, and their corresponding QQ plots closely followed the theoretical expectation, indicating a lower number of strongly associated SNPs (Figure 7).

A total of 25 significant SNP markers (*p* < 0.001) were identified across 16 chromosomes (Table 5). For DBH growth, five highly significant loci were detected on chromosomes 6, 10, and 11, with the strongest association observed at locus 10_31605746 (*p* = 2.18 × 10^−4^). In addition, two adjacent loci on chromosome 10—10_68523476 and 10_68494457—also exhibited strong associations. These three loci may reside within the same linkage block, suggesting the presence of key functional genes involved in DBH regulation. For height growth, only two significant loci (*p* < 0.0001) were identified, with 9_55280744 (*p* = 5.40 × 10^−6^) showing the strongest association, potentially representing a major-effect locus influencing height variation. Branch number exhibited the most extensive association pattern, with 18 highly significant SNPs. Notably, loci 17_69375264 (*p* = 1.03 × 10^−5^) and 12_60323301 (*p* = 2.13 × 10^−5^) displayed the strongest correlations, and are likely involved in axillary bud differentiation or branching-related regulatory pathways. These associations were distributed across chromosomes 1–8, 10–12, and 15–19, supporting the polygenic architecture underlying variation in branch number.

### 2.6. Construction of DNA Fingerprints

Genotyping was conducted using the 13 selected core SNP markers, resulting in a 13 × 197 genotype matrix. For the remaining 100 accessions without resequencing data, locus-specific primers were designed to amplify the corresponding SNP regions via PCR. Sanger sequencing of qualified PCR products, followed by manual inspection of chromatograms, enabled accurate genotype calling, ultimately yielding a standardized 13-locus SNP genotype matrix. Genotype data from all 297 *Liriodendron* accessions were concatenated according to genomic coordinates to generate unique fingerprint codes. These codes, produced by sequentially combining genotypes at the 13 core loci, enabled precise individual-level discrimination. A comprehensive molecular fingerprinting system was established by integrating the 13 SNP markers with 34 phenotypic traits. Each sample’s QR code encapsulated its accession ID, SNP genotype profile, and phenotypic data (Table 6; Supplementary Table S1), thereby supporting efficient identification, traceability, and germplasm management.

## 3. Discussion

Recent advances in forest genetic improvement have underscored the need for systematic conservation and utilization of rare tree germplasm resources. This paradigm shift reflects both ecological imperatives and the demands of modern breeding programs, particularly for relict species with narrow natural distributions [29,30,31]. As a representative genus within the Magnoliaceae family, *Liriodendron* possesses considerable ecological, ornamental, and economic value, rendering genetic diversity assessment and germplasm identification key research priorities. Genetic diversity, a fundamental indicator of a species’ adaptive capacity, is shaped by factors such as genetic drift, natural selection, and gene flow [32]. To support the development of effective breeding strategies for *Liriodendron*, we first evaluated phenotypic variation among 297 accessions. Phenotypic traits, which reflect morphological-level genetic diversity, were assessed using coefficients of variation (CV), with higher values indicating greater variability in germplasm resources [33]. Analysis of key growth traits including annual mean diameter at breast height increment, annual height increment, and crown spread revealed consistently high coefficients of variation, demonstrating substantial genetic differentiation within the *Liriodendron* genus. This pronounced phenotypic variation not only establishes critical selection criteria for superior germplasm identification but also underscores the remarkable phenotypic plasticity of *Liriodendron* species in specific growth characteristics, thereby enhancing breeding potential. Notably, germplasm exhibiting greater crown dimensions shows particular suitability for landscape applications, while accessions with accelerated growth rates are ideally suited for timber plantation development [34]. Of particular significance is the exceptional variation observed in stem form and branching architecture traits, which directly determine crown structure and wood properties [35]. These findings provide valuable insights for optimizing silvicultural practices and informing strategic breeding programs for varietal improvement.

As a fundamental determinant of evolutionary resilience and adaptive capacity, genetic diversity provides an essential baseline for both germplasm conservation and breeding applications [22]. In this study, we conducted a genome-wide SNP analysis across 297 *Liriodendron* accessions representing three taxonomically distinct groups: *L. chinense*, *L. tulipifera*, and their interspecific hybrids. As the most abundant form of genomic variation, SNPs offer several advantages—including codominance, amplification stability, and high reproducibility—which make them particularly suitable for assessing genetic diversity [36]. In recent years, SNP-based approaches have been widely applied in plant genetics research, significantly advancing germplasm management and varietal conservation efforts [37]. From an initial pool of variants, 4204 high-quality SNPs were stringently selected, showing a non-uniform distribution across the genome with evident regional variation. These markers exhibited an average PIC of 0.159, a critical index for evaluating inter-accession polymorphism and supporting gene pool development and breeding acceleration [38]. Compared with those in other forest species, such as *Picea abies* (PIC ≈ 0.12) [39] and *Betula platyphylla* (He ≈ 0.141) [40], the polymorphism levels observed in *Liriodendron* were representative and suitable for germplasm evaluation. The overall observed heterozygosity (Ho = 0.203), expected heterozygosity (He = 0.154), and Shannon’s diversity index (H′ = 0.364) collectively indicated a substantial degree of genetic diversity within the sampled population. Notably, the *Liriodendron sino-americanum* group exhibited the highest diversity across all indices, likely resulting from the incorporation of biparental allelic variation through interspecific hybridization. This pattern is consistent with prior studies on interspecific heterosis in *Liriodendron sino-americanum* and may represent a broader evolutionary trend, as similar diversity-enhancing effects have been reported in other woody taxa, such as poplar hybrid systems [41].

Genetic structure reveals the distribution patterns of genetic diversity within and among populations, serving as an important indicator of a species’ adaptive potential to its environment [42]. To characterize the genetic structure of *Liriodendron* germplasm resources, we conducted a series of genetic variation analyses. Population structure analysis indicated that the optimal clustering occurred at K = 3, dividing the 297 accessions into three genetic groups. Both principal component analysis and hierarchical clustering produced consistent results, which aligned with our expectations. Notably, North American *L. tulipifera* exhibited a broader genetic distribution than *L. chinense* and *Liriodendron sino-americanum*, potentially due to higher genetic heterogeneity or more complex evolutionary lineages within its populations. Long et al. similarly reported that *L. tulipifera* harbors approximately 1.8 times the genetic diversity of *L. chinense*, [43] likely resulting from multiple contributing factors such as geographic isolation, restricted gene flow [44], and historical domestication bottlenecks [45]. In the K = 3 population structure simulation, most accessions displayed mixed ancestry components, indicating frequent gene flow among groups and leading to partial differentiation without complete population separation. This observation is consistent with findings from red-fruited *Ailanthus altissima* varieties [19]. Genome-wide association analysis integrating genomic and phenotypic datasets revealed 25 significantly associated SNPs corresponding to DBH, tree height, and branch number traits, with primary genomic distributions on chromosomes 10, 11, and 17. Notably, the most strongly associated SNPs for these respective traits were 10_31605746, 9_55280744, and 17_69375264. These findings not only elucidate the molecular basis of key phenotypic characteristics in *Liriodendron* but also provide reliable target loci for molecular marker-assisted selection. The identified SNPs facilitate early trait prediction at the seedling stage through genotyping, potentially shortening the breeding cycle and accelerating the development of superior cultivars [46]. This dual “structure-function” approach has demonstrated practical utility, as reported by Resende et al. in their landmark study on *Eucalyptus*, this dual structure–function approach has already proven its practical value in tree breeding [47].

Building upon our previous systematic evaluations of phenotypic traits, genetic diversity, population structure, and genome-wide association analyses of key trait-associated SNPs, we established a robust theoretical and data-driven foundation for DNA fingerprint development in *Liriodendron*. Although DNA fingerprinting has been widely applied in crops and woody shrubs [48,49], no such database previously existed for *Liriodendron* species. The selection of appropriate SNP markers is essential for developing a DNA fingerprint database. Using a stepwise additive algorithm, we identified 13 core SNP markers demonstrating high polymorphism (PIC = 0.34), balanced genotype distribution, broad genomic coverage (spanning 8 chromosomes), and excellent discriminative ability. After validation, these markers were used to establish a DNA fingerprint database for 297 Populus germplasm samples. This database enables precise germplasm identification, promotes the shift from conventional to precision breeding methods, and offers molecular tools for Populus germplasm evaluation and breeding. Moreover, these core SNP markers serve as key links between molecular assays and breeding applications, valuable tools for germplasm identification, genetic relationship studies, marker-assisted breeding, and genetic map development [50,51,52]. As the first comprehensive effort to characterize *Liriodendron* germplasm at both the phenotypic and molecular levels, our findings offer critical scientific support for the conservation, precise identification, and innovative utilization of these valuable genetic resources.

## 4. Materials and Methods

### 4.1. Research on Germplasm Materials and Phenotypic Traits

A total of 297 *Liriodendron* germplasm accessions—including *L. chinense*, *L. tulipifera*, and their hybrid *Liriodendron × sinoamericanum*—were collected in July 2023 from the *Liriodendron* Germplasm Repository located at Xiashu Forest Farm (32°10′19.67″ N, 119°11′51.14″ E), a field station affiliated with Nanjing Forestry University (detailed metadata are provided in Supplementary Table S2). Fresh leaves or buds were immediately flash-frozen in liquid nitrogen and stored at −80 °C until DNA extraction. At the same time, Standardized protocols were implemented to measure phenotypic data for 34 traits spanning five key categories: growth parameters, branching architecture, leaf morphology, floral characteristics, and phenological phases (Table 7).

This study employed single nucleotide polymorphism (SNP) markers for experimental analysis. SNP markers were selected for germplasm identification and fingerprinting development due to their three fundamental advantages: genome-wide distribution, high polymorphism rates, and exceptional molecular stability. Whole-genome resequencing data from 197 representative accessions were used as the primary dataset for SNP discovery, population analysis, and fingerprinting development. Based on the core SNP markers identified from these data, SNP-specific primers were designed and validated via Sanger sequencing in an additional 100 accessions. All analyses were conducted using the reference genome assembly “Lchi1.0.a2_maker_aug.cds.filter.HCH.fasta,” developed and maintained by our research team.

### 4.2. Phenotypic Trait Data Processing and Evaluation

We quantitatively scored 34 traits across 297 *Liriodendron* germplasm accessions (Supplementary Table S3) and recorded all data uniformly using standardized protocols (Supplementary Table S4). Quantitative traits were processed through numerical coding using Microsoft Excel 2010, while descriptive traits were categorically classified. Phenotypic frequency distributions were analyzed for each trait, with coefficients of variation (CV = [SD/Mean] × 100%) and genetic diversity indices (H’ = −Σ[Pᵢ × lnPᵢ]) [53] calculated. Subsequent multivariate analyses included Pearson correlation and principal component analysis (PCA) performed in SPSS 24.0, hierarchical clustering conducted in Origin 2021(9.8) and comprehensive evaluation through membership function analysis to generate D-values for germplasm scoring.

### 4.3. DNA Extraction

Genomic DNA was extracted using the Tiangen Plant Genome DNA Kit. DNA integrity was verified by 1% agarose gel electrophoresis, with concentration and purity measured by NanoDrop spectrophotometry to ensure A260/A280 ratios between 1.7 and 1.9 and concentrations >50 ng/μL.

### 4.4. SNP Marker Screening and Sanger Sequencing Validation

#### 4.4.1. Quality SNPs Screening

Whole-genome resequencing (WGS) data from 197 *Liriodendron* specimens were initially quality-checked with FastQC, followed by alignment to the reference genome (Lchi1.0.a2_maker_aug.cds.filter.HCH.fasta) using BWA. The resulting files were converted to BAM format using SAMtools, followed by sorting and duplicate removal to obtain the final BAM files for downstream analysis.

For SNP filtering, GATK (Genome Analysis Toolkit) was used with the following criteria: SNP marker meeting any of the criteria were filtered out: QD < 2.0|MQ < 40.0|FS > 60.0|SOR > 3.0|MQRankSum < −12.5|ReadPosRankSum < −8.0. Subsequently, VCFtools v0.1.16 was applied for additional filtering: Genotype missing rate < 20%|MAF > 0.05|HWE *p* < 0.0001|Biallelic SNPs only. Finally, strict filtering was performed to remove: Genotype quality (GQ) < 30, SNPs with >1% missing genotypes. Sites with average depth <3× or outside the 3–100× range. This process yielded high-quality SNP marker for further analysis.

#### 4.4.2. Core SNPs Identification

A greedy feature increment algorithm was employed to identify the minimal set of core SNP markers. Initially, the genotype field (GT) was extracted from the VCF file using the scikit-allel package, and the genotypes at each SNP locus were standardized across all samples using the codes “0/0”, “0/1”, and “1/1”, resulting in a sample × locus genotype matrix. Genotype fingerprint codes were then generated by concatenating the genotypes of each sample for a given candidate SNP combination. In each iteration, the uniqueness of the fingerprint codes was assessed, and the most informative SNP site was progressively added until all 197 samples were assigned fully unique, non-redundant fingerprint codes. This procedure strictly adhered to the principle of minimizing the combination size to determine the smallest possible core SNP set.

#### 4.4.3. Sanger Sequencing Validation

To validate the accuracy and reproducibility of the selected core SNPs, specific primers were designed for each of the 13 core SNP markers. Eight representative samples were then subjected to PCR amplification followed by agarose gel electrophoresis analysis. The PCR amplification conditions and cycling protocol were as follows: The amplification was performed in a 20 μL reaction system containing 10 μL of 2× Rapid Taq Master Mix, 7 μL nuclease-free ddH_2_O, 1 μL template DNA (200 ng/μL), and 1 μL each of forward and reverse primers (300 ng/μL). The thermal cycling protocol consisted of an initial denaturation at 94 °C for 5 min, followed by 35 cycles of denaturation at 94 °C for 30 s, annealing at 58 °C for 30 s, and extension at 72 °C for 1 min, with a final extension at 72 °C for 5 min and hold at 16 °C.

For genotyping accuracy verification, randomly selected core markers and their representative genotyped samples were analyzed using Sanger sequencing.

### 4.5. Genetic Diversity Analysis

Genetic diversity analysis was performed using high-quality SNP data in the R environment by calculating seven polymorphism indices: allele number (Na), effective allele number (Ne), observed heterozygosity (Ho), expected heterozygosity (He), polymorphic information content (PIC), Shannon’s diversity index (H′), and Nei’s genetic diversity index (Nei). To evaluate genetic structure and population differentiation, principal component analysis (PCA), cluster analysis, linkage disequilibrium (LD) analysis, and population structure analysis were conducted. A custom Python script was used to parse VCF files and construct a numerical genotype matrix, with PCA implemented using the scikit-learn module. Euclidean genetic distances between samples were calculated using the pdist function in SciPy 1.15.0 followed by average-linkage hierarchical clustering and dendrogram visualization. For LD analysis, the top 1000 markers from the chromosome with the highest number of SNPs were selected, and pairwise LD (*r*^2^) values were calculated using the Rogers–Huff method in scikit-allel, incorporating physical distance data. Population structure was inferred for all 197 individuals using STRUCTURE 2.3, based on a Bayesian clustering approach. Trait–SNP associations were evaluated through a genome-wide association study (GWAS) using a general linear model (GLM), in which the genotype matrix was subjected to linear regression. The GLM was defined as:

Y = Xβ + ε

here Y: Phenotypic trait vector (*n* × 1), X: Design matrix including the intercept and SNP genotype data (*n* × *p*), β: Fixed-effect parameter vector (*p* × 1), representing SNP effect sizes, ε: Residual term, assumed to follow ε ~ *n*(0, σ^2^)

### 4.6. DNA Fingerprinting Construction

Genotype information (GT field) for the core SNP markers was extracted from the VCF files and converted into a biallelic format. For each sample, genotype strings of the core markers were concatenated in a fixed genomic order—based on chromosome number and physical position—to generate complete genotype fingerprint codes.

QR code images were generated using Python 3.11 qrcode library by encoding both the fingerprint codes and associated trait data of each *Liriodendron* accession. The resulting QR codes were embedded into an Excel-based fingerprint map using the openpyxl package, producing a visualized “one-code-one-image” format for all accessions.

## 5. Conclusions

This study systematically characterized *Liriodendron* germplasm resources through integrated phenotypic and molecular analyses. Phenotypic evaluation of key traits (DBH, tree height, branch number, and crown width) revealed significant variation and trait correlations within the genus, elucidating fundamental germplasm structures that inform selective breeding strategies and superior germplasm identification, thereby enhancing breeding efficiency. Molecular analyses employing high-quality SNPs clarified the genetic diversity and population structure of *Liriodendron* species. Further, we developed a core SNP-based fingerprinting system that uniquely identifies all accessions through QR-coded digital markers, enabling efficient germplasm management and traceability. These integrated approaches provide robust technical support for *Liriodendron* germplasm identification, evaluation, and conservation, and favor advancing breeding process for this genus.

## Figures and Tables

**Table 1:** Analysis of genetic variation in 34 traits across 297 germplasm samples.

Phenotypic Trait	Maximum	Minimum	Mean	Standard Deviation (SD)	Coefficient of Variation (%)	Shannon’s Diversity Index (H’)
DBH annual growth	2.85	0.40	1.62	0.43	26.57	5.23
Height annual growth	1.63	0.32	1.22	0.29	24.25	4.72
Branch number	48	1	19.63	7.40	37.68	3.34
Under-branch height	3	1	2.34	0.69	29.45	0.98
Crown shape	3	1	2.41	0.89	37.03	0.74
Crown width	3	1	1.60	0.61	38.46	0.89
Lenticel	2	1	1.81	0.39	21.82	0.49
Stem form	5	1	2.58	1.20	46.58	1.41
Bark fissuring	3	1	1.93	0.27	14.27	0.29
Bark color	2	1	1.33	0.47	35.41	0.63
Branch diameter	3	1	1.65	0.67	40.85	0.96
Branch density	3	1	1.87	0.76	40.85	1.07
Epidermis color	2	1	1.97	0.16	8.22	0.12
Juvenile leaf color	3	3	3	0	0	0
Mature leaf color	3	3	3	0	0	0
Summer leaf color	4	2	3.47	0.51	14.59	0.71
Autumn leaf color	4	1	1.90	0.68	35.56	0.74
Leaf shape	3	1	1.94	0.40	20.73	0.55
Number of leaf lobes	3	1	1.94	0.40	20.73	0.55
Leaf lobe depth	3	1	2.02	0.49	24.39	0.72
Central lobe angle	3	1	2.13	0.48	22.47	0.69
Leaf margin	1	1	1	0	0	0
Leaf base shape	6	2	2.03	0.35	17.26	0.09
Bud burst timing	3	1	2.17	0.74	33.88	1.05
Flowering period	3	1	1.98	0.61	30.68	0.91
Leaf coloration period	3	1	2.07	0.59	28.49	0.88
Leaf color duration	3	1	2.10	0.58	27.59	0.87
Standard term	3	1	2.18	0.60	27.70	0.90
Leaf abscission date	3	1	2.11	0.60	28.58	0.91
Corolla shape	3	2	2.06	0.23	11.31	0.22
Inner tepal color	2	2	2	0	0	0
Tip recurvature	2	1	1.21	0.40	33.57	0.51
Floral striping	1	1	1	0	0	0
Color striping	1	1	1	0	0	0

**Table 2:** Core SNP Marker and primer information.

Chromosome	Position	Forward_Primer	Reverse_Primer	PIC
chr7	54379502	TTGCTCCCCCATAACCTG	ATGCTAATCTATGCCTTGGTC	0.3737
chr9	25701542	CGATCATGAATTTTCTACCCCT	AGCTCCCCAAGTATATCCCA	0.3702
chr1	108063211	ACATGATAGGAAAGCCCGAC	TGCAGTAAACCCAAGGCAAC	0.3686
chr1	19625439	AGACTAATTCCTTCCGGCTA	CGAGACTCTACTTTTCGGAT	0.3666
chr13	360931	GTCGTCTTTCCCATTCGAT	ATTTTACCAAGCAATGCCTC	0.3655
chr2	75819730	TACAGGAGCAAATCATCCAG	CATTAGGCAGACTCAATCCA	0.3647
chr2	72967413	ATGTAATCCCGTTTACTCCC	TAAGATCAGGCCAAGTGCAT	0.3496
chr15	71449735	AAAAGCAAATTCGCGGAG	TTTCGATGCTACCGTGGACA	0.3496
chr7	29021	AGCCATTTTAATGATCCACAC	ACTAGCCTCAATAAGAATGC	0.3280
chr10	67277714	ATGTTTGGGAGAAATCCAGT	CCGCTCATGGTTTTAATCGTT	0.3280
chr19	9446947	CAATCAGGTAATAGGCTCGT	AGAAGCCGTTGATAGATCCA	0.3206
chr9	55279902	ATGAATGGGCTACACCAC	AATACATGAAATTCAGCAACA	0.2965
chr15	71464364	GTATATCCACCCCGTCCA	CCTTCCATCTAGTGCGCTTT	0.2907

**Table 3:** Three Genotypes at One SNP Locus in Three Samples and Their Sequencing Maps.

Sample ID	SNP Sites	Genotype	Sequencing Chromatogram
S_BK1_163	7:54379502	0/0	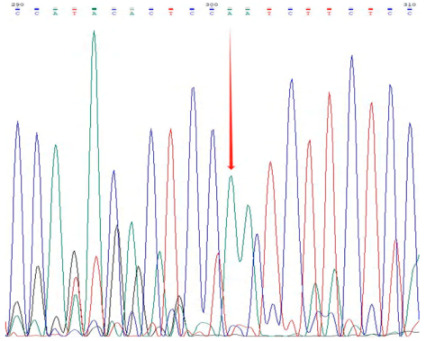
BK1_S_133	7:54379502	0/1	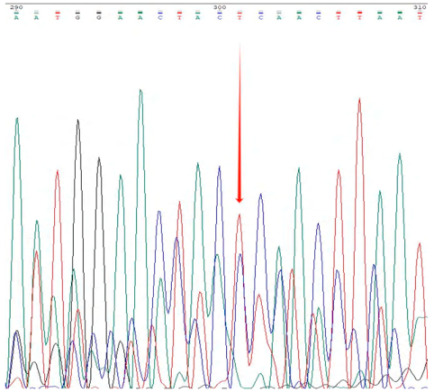
BK1_S_27	7:54379502	1/1	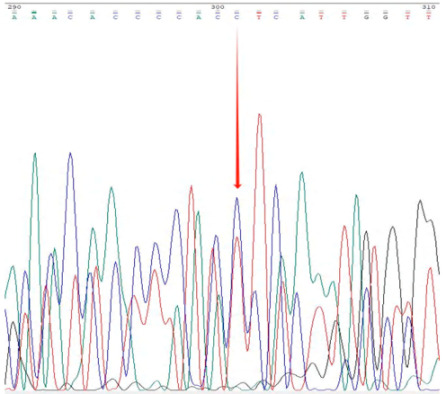

**Table 4:** Genetic diversity analysis of different *Liriodendron* species.

Class	Na	Ne	Ho	He	PIC	H’	Nei
*Liriodendron sino-americanum*	2.00	1.23	0.20	0.18	0.14	0.46	0.18
*L. tulipifera*	1.54	1.23	0.19	0.15	0.10	0.35	0.15
*L.chinense*	1.36	1.23	0.21	0.13	0.08	0.29	0.13

**Table 5:** Significantly associated SNPs with phenotypic traits identified.

Trait	SNP ID	Chr	Pos	*p*	Significance
DBH growth per year	10_31605746	10	31,605,746	0.00021	*p* < 0.001
DBH growth per year	10_68523476	10	68,523,476	0.00028	*p* < 0.001
DBH growth per year	10_68494457	10	68,494,457	0.00045	*p* < 0.001
DBH growth per year	11_20535981	11	20,535,981	0.00062	*p* < 0.001
DBH growth per year	6_25999454	6	25,999,454	0.00095	*p* < 0.001
DBH growth per year	9_55280744	9	55,280,744	0.000005	*p* < 0.0001
DBH growth per year	11_20133806	11	20,133,806	0.00005	*p* < 0.0001
Number of branches	17_69375264	17	69,375,264	0.00001	*p* < 0.0001
Number of branches	12_60323301	12	60,323,301	0.00002	*p* < 0.0001
Number of branches	17_69369773	17	69,369,773	0.00004	*p* < 0.0001
Number of branches	19_32659357	19	32,659,357	0.00004	*p* < 0.0001
Number of branches	8_38736461	8	38,736,461	0.00007	*p* < 0.0001
Number of branches	4_64433608	4	64,433,608	0.0001	*p* < 0.001
Number of branches	6_70857940	6	70,857,940	0.0001	*p* < 0.001
Number of branches	3_7388221	3	7,388,221	0.0001	*p* < 0.001
Number of branches	5_32339010	5	32,339,010	0.00012	*p* < 0.001
Number of branches	10_68510137	10	68,510,137	0.00014	*p* < 0.001
Number of branches	6_22671960	6	22,671,960	0.00016	*p* < 0.001
Number of branches	10_68502860	10	68,502,860	0.00019	*p* < 0.001
Number of branches	1_7306959	1	7,306,959	0.00028	*p* < 0.001
Number of branches	15_62197488	15	62,197,488	0.00033	*p* < 0.001
Number of branches	4_13788354	4	13,788,354	0.00039	*p* < 0.001
Number of branches	1_96218257	1	96,218,257	0.00043	*p* < 0.001
Number of branches	7_26406718	7	26,406,718	0.00045	*p* < 0.001
Number of branches	11_18761672	11	18,761,672	0.00045	*p* < 0.001

**Table 6:** Examples of DNA fingerprinting and molecular ID codes for part of *Liriodendron* germplasm.

Sample ID	SNP Fingerprint Code	QR Code	Sample ID	SNP Fingerprint Code	QR Code
BK1_H_117	1/10/10/1.0/0.0/11/1.0/0.0/00/10/0...	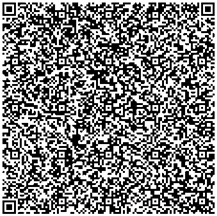	S_BK1_161	0/10/10/00/10/01/10/11/10/00/0...	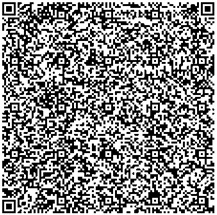
BK1_H_118	0/0.1/10/1.0/0.1/11/10/10/00/10/0...	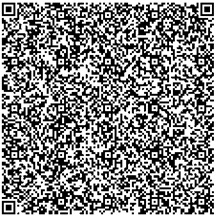	S_BK1_162	1/11/10/00/10/0.0/0.0/00/10/10/1...	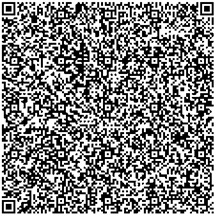
BK1_H_120	1/10/10/1.0/0.1/10/10/10/00/00/0...	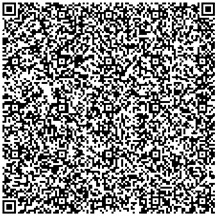	S_BK1_163	1/10/10/00/10/00/10/00/10/10/0...	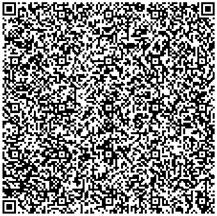

**Table 7:** Investigated Phenotypic Traits in *Liriodendron* spp.

	Classification	Phenotypic Trait
1	Growth Traits	DBH annual growth
2		Height annual growth
3		Branch number
4		Under-branch height
5		Crown shape
6		Crown width
7		Stem form
8		Lenticel
9		Bark fissuring
10		Bark color
11	Branch Traits	Branch density
12		Branch diameter
13		Epidermis color
14	Leaf Traits	Juvenile leaf color
15		Mature leaf color
16		Summer leaf color
17		Autumn leaf color
18		Leaf shape
19		Number of leaf lobes
20		Leaf lobe depth
21		Central lobe angle
22		Leaf margin
23		Leaf base shape
24	Flower Traits	Corolla shape
25		Inner tepal color
26		Tip recurvature
27		Floral striping
28		Color striping
29	Phenology	Bud burst timing
30		Flowering period
31		Leaf coloration period
32		Leaf color duration
33		Standard term
34		Leaf abscission date

## Data Availability

The research data supporting the findings of this study will be made available upon reasonable request. The necessary data is supplemented in the Supplementary file.

## Supplementary Materials

The following supporting information can be downloaded at: https://www.mdpi.com/article/10.3390/plants14172626/s1, Table S1: DNA Fingerprinting Profiles and Molecular IDs for 297 *Liriodendron* Accessions; Table S2: The Codes and Species attribution of 297 *Liriodendron* Germplasm Accessions; Table S3: Scoring Table for 34 Morphological Traits; Table S4: Data Collection Sheets for 34 Morphological Traits in 297 *Liriodendron* Accessions.
