# Supplementary material for: Comprehensive Evaluation and DNA Fingerprints of Liriodendron Germplasm Accessions Based on Phenotypic Traits and SNP Markers
Source: Plants (Basel). 2025 Aug 23;14(17):2626. doi: 10.3390/plants14172626 (PMC12430399; doi:10.3390/plants14172626)
Supplement: Supplementary file 1 [file plants-14-02626-s001.zip › Table S2.pdf]

Supplementary Table S2. The Codes and Species attribution of297 *Liriodendron* Germplasm Accessions

No	Code	Species of tress	No	Code	Species of tress	No	Code	Species of tress
1	BK1_H_117	*Liriodendron sino-americanum*	101	M_SZ1_195	*Liriodendron sino-americanum*	201	BK-13	*L. tulipifera*
2	BK1_H_118	*Liriodendron sino-americanum*	102	M_SZ1_205	*Liriodendron sino-americanum*	202	LYS-17	*L. tulipifera*
3	BK1_H_120	*Liriodendron sino-americanum*	103	M_SZ1_206	*Liriodendron sino-americanum*	203	LYS-18	*L. tulipifera*
4	BK1_H_121	*Liriodendron sino-americanum*	104	M_SZ1_207	*Liriodendron sino-americanum*	204	LYS-19	*L. tulipifera*
5	BK1_H_231	*Liriodendron sino-americanum*	105	M_SZ1_208	*Liriodendron sino-americanum*	205	NK-20	*L. tulipifera*
6	BK1_H_46	*Liriodendron sino-americanum*	106	M_SZ1_209	*Liriodendron sino-americanum*	206	NK-23	*L. tulipifera*
7	BK1_H_47	*Liriodendron sino-americanum*	107	M_SZ1_83	*Liriodendron sino-americanum*	207	NK-25	*L. tulipifera*
8	BK1_H_99	*Liriodendron sino-americanum*	108	M_S_105	*Liriodendron sino-americanum*	208	ZZY-27	*L. tulipifera*
9	BK1_P_270	*Liriodendron sino-americanum*	109	M_S_167	*Liriodendron sino-americanum*	209	ZZY-28	*L. tulipifera*
10	BK1_S_132	*Liriodendron sino-americanum*	110	M_S_201	*Liriodendron sino-americanum*	210	XY-31	*L. chinense*
11	BK1_S_133	*Liriodendron sino-americanum*	111	M_S_202	*Liriodendron sino-americanum*	211	HS-38	*L. chinense*
12	BK1_S_134	*Liriodendron sino-americanum*	112	M_S_204	*Liriodendron sino-americanum*	212	HS-40	*L. chinense*
13	BK1_S_135	*Liriodendron sino-americanum*	113	M_S_60	*Liriodendron sino-americanum*	213	HS-42	*L. chinense*
14	BK1_S_136	*Liriodendron sino-americanum*	114	M_WYS1_248	*Liriodendron sino-americanum*	214	SN-43	*L. chinense*
15	BK1_S_137	*Liriodendron sino-americanum*	115	M_WYS1_50	*Liriodendron sino-americanum*	215	SN-44	*L. chinense*
16	BK1_S_138	*Liriodendron sino-americanum*	116	M_WYS1_89	*Liriodendron sino-americanum*	216	WYS-46	*L. chinense*
17	BK1_S_239	*Liriodendron sino-americanum*	117	N1_J1_147	*Liriodendron sino-americanum*	217	WYS-48	*L. chinense*
18	BK1_S_240	*Liriodendron sino-americanum*	118	N1_J1_189	*Liriodendron sino-americanum*	218	WYS-49	*L. chinense*
19	BK1_S_26	*Liriodendron sino-americanum*	119	N1_J1_190	*Liriodendron sino-americanum*	219	WYS-50	*L. chinense*
20	BK1_S_27	*Liriodendron sino-americanum*	120	N1_J1_237	*Liriodendron sino-americanum*	220	LYS-51	*L. tulipifera*
21	BK1_S_69	*Liriodendron sino-americanum*	121	N1_J1_238	*Liriodendron sino-americanum*	221	XN-52	*L. chinense*
22	BK1_S_71	*Liriodendron sino-americanum*	122	N1_J1_79	*Liriodendron sino-americanum*	222	XN-56+	*L. chinense*
23	C1_P_271	*Liriodendron sino-americanum*	123	N1_J1_80	*Liriodendron sino-americanum*	223	EX-58	*L. chinense*
24	FY1_P_272	*Liriodendron sino-americanum*	124	N1_P_277	*Liriodendron sino-americanum*	224	EX-60	*L. chinense*
25	H_L_155	*Liriodendron sino-americanum*	125	N1_SN1_130	*Liriodendron sino-americanum*	225	EX-61	*L. chinense*
26	H_L_157	*Liriodendron sino-americanum*	126	N1_SN1_131	*Liriodendron sino-americanum*	226	SY-69	*L. chinense*
27	H_L_235	*Liriodendron sino-americanum*	127	N1_SN1_210	*Liriodendron sino-americanum*	227	SY-70	*L. chinense*
28	H_L_236	*Liriodendron sino-americanum*	128	N1_SN1_211	*Liriodendron sino-americanum*	228	YY-75	*L. chinense*
29	H_L_84	*Liriodendron sino-americanum*	129	N1_SN1_33	*Liriodendron sino-americanum*	229	YY-76	*L. chinense*
30	H_L_85	*Liriodendron sino-americanum*	130	N1_SN1_35	*Liriodendron sino-americanum*	230	LS-81	*L. chinense*
31	H_L_86	*Liriodendron sino-americanum*	131	N1_SN1_36	*Liriodendron sino-americanum*	231	LS-82	*L. chinense*
32	H_P_273	*Liriodendron sino-americanum*	132	N1_SN1_37	*Liriodendron sino-americanum*	232	LS-85	*L. chinense*
33	J1_L_123	*Liriodendron*	133	N1_SZ1_1	*Liriodendron*	233	SN-86	*L. chinense*

		sino-americanum			*sino-americanum*			
34	J1_L_125	*Liriodendron sino-americanum*	134	N1_SZ1_2	*Liriodendron sino-americanum*	234	SN-87	*L. chinense*
35	J1_L_129	*Liriodendron sino-americanum*	135	N1_SZ1_4	*Liriodendron sino-americanum*	235	BM*C1-W1	*Liriodendron sino-americanum*
36	J1_L_51	*Liriodendron sino-americanum*	136	N1_S_143	*Liriodendron sino-americanum*	236	BM*C1-W2	*Liriodendron sino-americanum*
37	J1_L_96	*Liriodendron sino-americanum*	137	N1_S_144	*Liriodendron sino-americanum*	237	N1*S-U1	*Liriodendron sino-americanum*
38	J1_L_97	*Liriodendron sino-americanum*	138	N1_S_145	*Liriodendron sino-americanum*	238	H*F1-W12	*Liriodendron sino-americanum*

Continued Table S1

No	Code	Species of tress	No	Code	Species of tress	No	Code	Species of tress
39	J1_L_98	*Liriodendron sino-americanum*	139	N1_S_217	*Liriodendron sino-americanum*	239	M*W-U2	*Liriodendron sino-americanum*
40	J1_P_274	*Liriodendron sino-americanum*	140	N1_S_218	*Liriodendron sino-americanum*	240	S*F2-W4	*Liriodendron sino-americanum*
41	L_C1_104	*Liriodendron sino-americanum*	141	N1_S_38	*Liriodendron sino-americanum*	241	C4-W9	*L. chinense*
42	L_C1_139	*Liriodendron sino-americanum*	142	N1_S_39	*Liriodendron sino-americanum*	242	AN-1-W14	*L. tulipifera*
43	L_C1_140	*Liriodendron sino-americanum*	143	N1_S_40	*Liriodendron sino-americanum*	243	XY-1-Z1	*L. chinense*
44	L_C1_141	*Liriodendron sino-americanum*	144	N1_S_41	*Liriodendron sino-americanum*	244	BK-4-U4	*L. tulipifera*
45	L_C1_142	*Liriodendron sino-americanum*	145	N1_S_8	*Liriodendron sino-americanum*	245	WYS-1-U7	*L. chinense*
46	L_C1_198	*Liriodendron sino-americanum*	146	SN1_P_279	*Liriodendron sino-americanum*	246	C5-W6	*L. chinense*
47	L_C1_28	*Liriodendron sino-americanum*	147	SZ1_P_280	*Liriodendron sino-americanum*	247	MB-10-U8	*L. tulipifera*
48	L_FY1_107	*Liriodendron sino-americanum*	148	S_BK1_159	*Liriodendron sino-americanum*	248	BK3-U9	*L. tulipifera*
49	L_FY1_108	*Liriodendron sino-americanum*	149	S_BK1_160	*Liriodendron sino-americanum*	249	BK3-U10	*L. tulipifera*
50	L_FY1_109	*Liriodendron sino-americanum*	150	S_BK1_161	*Liriodendron sino-americanum*	250	MSL-1-U11	*L. tulipifera*
51	L_FY1_12	*Liriodendron sino-americanum*	151	S_BK1_162	*Liriodendron sino-americanum*	251	MSL-1-U12	*L. tulipifera*
52	L_FY1_13	*Liriodendron sino-americanum*	152	S_BK1_163	*Liriodendron sino-americanum*	252	MSL-1-U13	*L. tulipifera*
53	L_FY1_224	*Liriodendron sino-americanum*	153	S_BK1_180	*Liriodendron sino-americanum*	253	C2-W7	*L. chinense*
54	L_FY1_74	*Liriodendron sino-americanum*	154	S_BK1_181	*Liriodendron sino-americanum*	254	D1ZH1	*Liriodendron sino-americanum*
55	L_J1_148	*Liriodendron sino-americanum*	155	S_BK1_91	*Liriodendron sino-americanum*	255	D1U1	*Liriodendron sino-americanum*
56	L_J1_149	*Liriodendron sino-americanum*	156	S_BK1_92	*Liriodendron sino-americanum*	256	D1Z1	*Liriodendron sino-americanum*
57	L_J1_150	*Liriodendron sino-americanum*	157	S_BK1_93	*Liriodendron sino-americanum*	257	D1Z4	*Liriodendron sino-americanum*
58	L_J1_212	*Liriodendron sino-americanum*	158	S_L_100	*Liriodendron sino-americanum*	258	D1U2	*Liriodendron sino-americanum*
59	L_J1_213	*Liriodendron sino-americanum*	159	S_L_101	*Liriodendron sino-americanum*	259	D1U3	*Liriodendron sino-americanum*
60	L_J1_214	*Liriodendron sino-americanum*	160	S_L_111	*Liriodendron sino-americanum*	260	D1U4	*Liriodendron sino-americanum*
61	L_J1_215	*Liriodendron*	161	S_L_112	*Liriodendron*	261	D1ZH6	*Liriodendron*

		sino-americanum			*sino-americanum*			*sino-americanum*
62	L_J1_216	*Liriodendron sino-americanum*	162	S_L_113	*Liriodendron sino-americanum*	262	D1ZH3	*Liriodendron sino-americanum*
63	L_J1_45	*Liriodendron sino-americanum*	163	S_L_22	*Liriodendron sino-americanum*	263	D1ZH8	*Liriodendron sino-americanum*
64	L_J1_65	*Liriodendron sino-americanum*	164	S_L_226	*Liriodendron sino-americanum*	264	D2U3	*Liriodendron sino-americanum*
65	L_J1_67	*Liriodendron sino-americanum*	165	S_L_227	*Liriodendron sino-americanum*	265	D2U4	*Liriodendron sino-americanum*
66	L_J1_68	*Liriodendron sino-americanum*	166	S_L_228	*Liriodendron sino-americanum*	266	D2U6	*Liriodendron sino-americanum*
67	L_P_275	*Liriodendron sino-americanum*	167	S_L_229	*Liriodendron sino-americanum*	267	D2ZH1	*Liriodendron sino-americanum*
68	L_S_14	*Liriodendron sino-americanum*	168	S_L_230	*Liriodendron sino-americanum*	268	D2ZH2	*Liriodendron sino-americanum*
69	L_S_15	*Liriodendron sino-americanum*	169	S_L_24	*Liriodendron sino-americanum*	269	D2ZH3	*Liriodendron sino-americanum*
70	L_S_17	*Liriodendron sino-americanum*	170	S_L_25	*Liriodendron sino-americanum*	270	D2ZH8	*Liriodendron sino-americanum*
71	L_S_18	*Liriodendron sino-americanum*	171	S_N1_170	*Liriodendron sino-americanum*	271	D2U7	*Liriodendron sino-americanum*
72	L_S_186	*Liriodendron sino-americanum*	172	S_N1_171	*Liriodendron sino-americanum*	272	D2U8	*Liriodendron sino-americanum*
73	L_S_187	*Liriodendron sino-americanum*	173	S_N1_172	*Liriodendron sino-americanum*	273	D2U12	*Liriodendron sino-americanum*
74	L_S_19	*Liriodendron sino-americanum*	174	S_N1_173	*Liriodendron sino-americanum*	274	D2W3	*Liriodendron sino-americanum*
75	L_S_249	*Liriodendron sino-americanum*	175	S_N1_175	*Liriodendron sino-americanum*	275	D2W4	*Liriodendron sino-americanum*
76	L_S_29	*Liriodendron sino-americanum*	176	S_N1_176	*Liriodendron sino-americanum*	276	D2Z5	*Liriodendron sino-americanum*
77	L_S_30	*Liriodendron sino-americanum*	177	S_N1_59	*Liriodendron sino-americanum*	277	D2Z6	*Liriodendron sino-americanum*

Continued Table S1

No	Code	Species of tress	No	Code	Species of tress	No	Code	Species of tress
78	L_S_31	*Liriodendron sino-americanum*	178	S_P_278	*Liriodendron sino-americanum*	278	D2ZH4	*Liriodendron sino-americanum*
79	L_S_32	*Liriodendron sino-americanum*	179	S_Z1_158	*Liriodendron sino-americanum*	279	D2U13	*Liriodendron sino-americanum*
80	L_YY1_106	*Liriodendron sino-americanum*	180	S_Z1_20	*Liriodendron sino-americanum*	280	D3U4	*Liriodendron sino-americanum*
81	L_YY1_164	*Liriodendron sino-americanum*	181	S_Z1_21	*Liriodendron sino-americanum*	281	D3U5	*Liriodendron sino-americanum*
82	L_YY1_165	*Liriodendron sino-americanum*	182	S_Z1_61	*Liriodendron sino-americanum*	282	D3Z1	*Liriodendron sino-americanum*
83	L_YY1_166	*Liriodendron sino-americanum*	183	S_Z1_62	*Liriodendron sino-americanum*	283	D3Z4	*Liriodendron sino-americanum*
84	L_YY1_220	*Liriodendron sino-americanum*	184	S_Z1_63	*Liriodendron sino-americanum*	284	D3Z6	*Liriodendron sino-americanum*
85	L_YY1_221	*Liriodendron sino-americanum*	185	WYS1_P_281	*Liriodendron sino-americanum*	285	D3Z7	*Liriodendron sino-americanum*
86	L_YY1_222	*Liriodendron sino-americanum*	186	YY1_P_282	*Liriodendron sino-americanum*	286	D3Z8	*Liriodendron sino-americanum*
87	L_YY1_223	*Liriodendron sino-americanum*	187	Z1_FY1_177	*Liriodendron sino-americanum*	287	D3Z9	*Liriodendron sino-americanum*
88	M_H_152	*Liriodendron sino-americanum*	188	Z1_FY1_178	*Liriodendron sino-americanum*	288	D3Z10	*Liriodendron sino-americanum*

89	M_H_153	*Liriodendron sino-americanum*	189	Z1_FY1_225	*Liriodendron sino-americanum*	289	D3ZH1	*Liriodendron sino-americanum*
90	M_H_154	*Liriodendron sino-americanum*	190	Z1_FY1_78	*Liriodendron sino-americanum*	290	D3ZH3	*Liriodendron sino-americanum*
91	M_H_233	*Liriodendron sino-americanum*	191	Z1_P_283	*Liriodendron sino-americanum*	291	D3ZH7	*Liriodendron sino-americanum*
92	M_H_44	*Liriodendron sino-americanum*	192	Z1_WYS1_102	*Liriodendron sino-americanum*	292	D3ZH10	*Liriodendron sino-americanum*
93	M_H_81	*Liriodendron sino-americanum*	193	Z1_WYS1_103	*Liriodendron sino-americanum*	293	D3W1	*Liriodendron sino-americanum*
94	M_H_82	*Liriodendron sino-americanum*	194	Z1_WYS1_114	*Liriodendron sino-americanum*	294	D4W1	*Liriodendron sino-americanum*
95	M_J1_252	*Liriodendron sino-americanum*	195	Z1_WYS1_115	*Liriodendron sino-americanum*	295	D4W2	*Liriodendron sino-americanum*
96	M_J1_57	*Liriodendron sino-americanum*	196	Z1_WYS1_116	*Liriodendron sino-americanum*	296	D4W5	*Liriodendron sino-americanum*
97	M_J1_94	*Liriodendron sino-americanum*	197	Z1_WYS1_168	*Liriodendron sino-americanum*	297	D4W6	*Liriodendron sino-americanum*
98	M_J1_95	*Liriodendron sino-americanum*	198	MSL-1	*L. tulipifera*			
99	M_P_276	*Liriodendron sino-americanum*	199	MSL-3	*L. tulipifera*			
100	M_SZ1_194	*Liriodendron sino-americanum*	200	BK-10	*L. tulipifera*			
